# Pre-operative hypoalbuminaemia predicts poor overall survival in rectal cancer: a retrospective cohort analysis

**DOI:** 10.1186/1472-6890-13-12

**Published:** 2013-04-16

**Authors:** Pramodh C Chandrasinghe, Dileepa S Ediriweera, Sumudu K Kumarage, Kemal I Deen

**Affiliations:** 1 Department of Surgery, North Colombo Teaching Hospital, Ragama, Sri Lanka

## Abstract

**Background:**

Serum albumin is a marker of nutrition and inflammation. It has recently emerged as a predictor of outcome after surgery for rectal cancer. Our aim was to evaluate if pre-operative serum albumin would predict survival after resection for rectal cancer.

**Method:**

226 Patients with rectal cancer of all stages undergoing resection with curative intent were studied. Kaplan-Meier curves analysed survival based on a pre-operative albumin level of <35 g/L vs. >35 g/L. We sought for significant associations of survival with age, sex, stage, tumour site, use of neoadjuvant chemoradiation, microscopic positive resection margins, differentiation, angio, peri-neural, and lymphovascular invasion using individual variable analysis. Multifactorial analysis was performed using type III analysis with Weibull hazard model and Cox-proportional hazard model. Significance was assigned to a P value <0.05.

**Results:**

Of 226 patients (median age- 59 years; range 19 – 88, Male - 54%), forty five (20%) had an albumin level < 35 g/L and was associated with a poor overall survival (P = 0.02). Mean survival in months for <35 g/L vs. >35 g/L was 64.7 (SE - 9.3) vs. 95.8 (SE – 7.0) and 5 year overall survival rates were 49% and 69%. Individual variable analysis revealed age, circumferential margin, stage, perineural, lympho-vascular and angio invasion to be also significant. With multifactorial analysis hypoalbuminaemia (HR = 0.58; 95% CI: 0.35 - 0.95, P = 0.03), advanced stage (HR = 2.0; 95% CI: 1.26 - 3.23, P < 0.01) and positive circumferential margin (HR = 2.2; 95% CI: 1.26 - 3.89, P < 0.01) remained significant.

**Conclusion:**

Preoperative hypoalbuminaemia is an independent risk factor for poor overall survival in rectal cancer. Advanced tumour stage and circumferential margin positivity were the other associations with poor survival.

## Background

Traditionally, serum albumin has been considered a marker of nutritional status [1,2]. In this regard, several studies have questioned its reliability as serum albumin concentration is affected by pro-inflammatory cytokines, stress hormone, metabolic rate, dehydration, hepatic and renal failure [3-5]. Currently, hypoalbuminaemia is used as a marker of inflammation, a predictor of outcome in post-surgical patients, and is associated with increased morbidity and mortality in patients having surgery for malignant disease [6,7]. The Glasgow Prognostic Score (GPS), which predicts outcome following surgery for colorectal cancer, also considers serum albumin level as an inflammatory marker rather than an indicator of nutrition [8]. Roxburgh et al. has reviewed the association between the systemic inflammatory response and survival in patients with cancer where hypoalbuminaemia was also recognised as a marker of inflammation [9]. Low serum albumin levels are known to contribute to post-operative complications such as anastomotic leakage, abdominal wound dehiscence and infection [10]. Although studies have established its predictive value in surgery for colon cancer [11,12], there is lack of such evidence in surgery for rectal cancer [13]. Evidence is emerging with regard to the significance of inflammatory response on survival in rectal cancer [14]. The aim of this study was to study the effect of preoperative serum albumin on survival following surgery for rectal cancer.

## Method

Two hundred and twenty six patients with rectal cancer, proven histologically, who had potentially curative resection at the university surgical unit between 1996 and 2010 were studied - patients with evidence of liver or kidney failure were excluded. Rectal cancer was defined as cancer within 12 cm from the anal verge – cancer from 0 to 6 cm was defined as distal rectal cancer. Pre-operative work up and management was standardized by protocol, and described in a previous publication [15]. Surgery was performed by the same team according to standard protocol and histological analysis of specimens was by a single pathologist. Data collection was pro-forma based in a continually updated prospective database, where, the pre-operative serum albumin level was entered in all patients. Post operative surveillance was carried out with clinical examination coupled with carcino-embryonic antigen (CEA) levels three monthly for the first 2 years and biannually for 3 years. A colonoscopy and computer tomographic evaluation of the abdomen and pelvis at 1, 3 and 5 years post operative was under taken. Study was approved by the ethics review committee at the University of Kelaniya medical school, Sri Lanka. Hypoalbuminaemia was defined as serum albumin level less than 35 g/L. Based on this value we defined two groups; pre-operative serum albumin less than 35 g/L and greater than 35 g/L. Furthermore, serially ascending values of serum albumin, in increments of 5 g/L, were analyzed to define a finite cut-off value. We compared overall survival, disease free survival at 5 years, thirty day mortality and post operative complications in the two groups. Initially, univariable analysis was performed to identify factors which affected survival; age, gender, site of the tumor (proximal rectum vs. distal rectum), pre-operative chaemoradiation, tumour stage, histological differentiation, circumferential and distal margin clearance, peri-neural, angio-invasion, and lymphovascular invasion. Post operative complications considered were wound infection, wound dehiscence and anastomotic leakage. Anastomotic leakage was diagnosed with clinical evidence of intra abdominal sepsis and ultrasound or CT evidence of intra peritoneal fluid. Cancers were staged according to the American Joint Committee on Cancer (AJCC) TNM classification. Microscopic tumour clearance of > 1 mm for circumferential margins was considered safe (R0). Distal margin clearance of >10mm for distal rectal cancers and > 50 mm for proximal cancers was considered as R0.

Data are expressed as median and range. A two sample test of proportions with a 95% confidence interval test was used to compare thirty day mortality and complication rates of those with serum albumin less than 35 g/L versus greater than 35 g/L. Survival analysis was performed using Kaplan Meier curves. Univariable analysis was performed using Kaplan-Meier analysis and Cox proportional hazard model on factors likely to affect survival, and we studied significant factors that affected survival by a type III analysis with a Weibull hazard model and two-way interaction terms. Hazard ratios (HR) for significant factors, identified through type III analysis, were computed using a Cox proportional hazard model. A P- value of less than 0.05 was regarded as significant. Statistical analysis was performed with the SAS/STAT statistical software (SAS system, version 9.0; SAS Institute, Cary, North Carolina).

## Results

**Figure 1:**
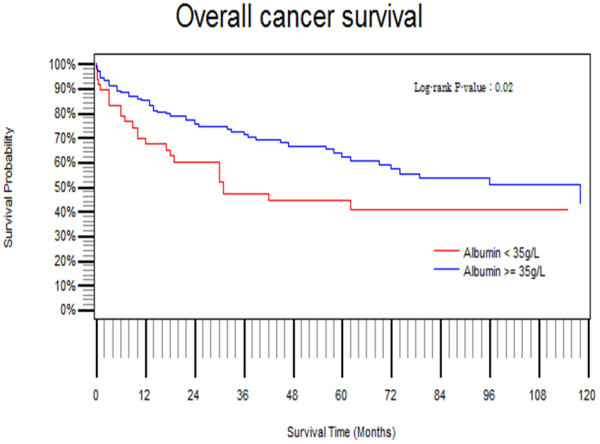
Overall survival comparison based on preoperative serum albumin level using Kaplan-Meier method.

123 (54%) Male and 103 female patients were studied. The median age of the population (n = 226) was 59 years (range 19 – 88 years). Median follow up was 36 months (range 10 – 160). Forty five patients (20%) had hypoalbuminaemia (serum albumin less than 3.5 g/L). We found that a serum albumin level of 35 g/L was the cut-off value at which most significant differences in survival emerged (Table 1). Overall survival following surgery for rectal cancer became significantly poor in patients having preoperative serum albumin less than 35 g/L compared with those with a serum albumin greater than 35 g/L (P = 0.02; Figure 1). Overall five year survival rates were 47% and 69% for the two groups respectively. The five year disease free survival rate in the hypoalbuminaemic group was 69.7% compared to 83% in those with a serum albumin above 35g/L (P = 0.02). Other factors which influenced survival significantly, using univariable analysis, were age, positive circumferential margin, peri-neural invasion, angio-invasion, lympho-vascular invasion, and advanced AJCC stage (Table 2). Multi factorial model type III analysis of effects revealed that hypo-albuminaemia (P = 0.002), a positive circumferential margin (P = 0.002), and AJCC stages III and IV compared with I and II (P = 0.003), were significant. However, when two-way interaction terms were added, using Weibull analysis, none was found to be significant (Table 3). Therefore hypoalbuminaemia, a positive circumferential margin, and advanced AJCC stage (III and IV) were identified as independent risk factors for poor survival following surgery for rectal cancer.

**Table 1:** Comparison of survival using Kaplan-Meier method based on serially ascending values of serum albumin

**Serum albumin level**	**5 year survival rates**	**P value**
<20 g/LVS. ≥20 g/L	90% VS. 60%	0.44
<25 g/LVS. ≥2 g/L	90% VS. 59%	0.10
<30 g/LVS. ≥30 g/L	43% VS. 63%	0.04^*^
<35 g/LVS. ≥35 g/L	49% VS. 69%	0.02^*^
<40 g/LVS. ≥40 g/L	58% VS. 62%	0.48
<45 g/LVS. ≥45 g/L	58% VS. 73%	0.17

* - significant value.

**Table 2:** Individual variable analysis for recognized determinants of survival for rectal cancer using Kaplan-Meier and Cox-proportional hazard model

**Factor total = 226(%)**	**Albumin < 35 g/L n = 45(%)**	**Albumin > 35 g/L n = 181(%)**	**P value**
Gender			0.09
Male	25(55%)	98(54%)	
Female	20(45%)	83(46%)	
Age	Continuos variable	Continuos variable	0.05
Site (Low < 6 cm)			0.79
Upper	27 (60%)	94 (52%)	
Lower	18 (40%)	87 (48%)	
Neoadjuvant CRT			0.90
Given	08 (18%)	68 (38%)	
Not given	37 (82%)	113 (62%)	
Tumour stage			0.002^*^
AJCC^+^	27 (60%)	91 (50%)	
AJCC III/IV	18 (40%)	90 (50%)	
Differentiation			0.18
Well	04 (9%)	26 (15%)	
Moderate	37 (82%)	133 (73%)	
Poor	04 (9%)	22 (12%)	
Circumferential margin			<0.001^*^
R0	35 (78%)	156 (86%)	
R1	10 (22%)	25 (14%)	
Distal margin			0.07
R0	40 (89%)	163 (90%)	
R1	05 (11%)	18 (10%)	
Peri-neural invasion	07 (16%)	18 (10%)	<0.001^*^
Angio invasion	05 (11%)	14 (8%)	<0.001^*^
Lympho-vascular invasion	08 (18%)	15 (8%)	<0.001^*^

+ American joint committee on cancer.

^*^Significant values.

**Table 3:** Two-way interaction terms for significant factors affecting survival using multifactorial analysis with a Weibull hazard model

**Factors**	**DF**	**Chi squared**	**P value**
Circumferential margin positivity	1	5.7647	0.01^*^
Serum albumin level	1	6.1745	0.01^*^
Circumferential margin positivity × serum albumin	1	0.9343	0.33
AJCC stage	1	7.8196	0.005^*^
Circumferential margin positivity × AJCC stage	1	1.6852	0.19
AJCC stage × serum albumin level	1	0.5503	0.45

× Interaction between two factors.

^*^Significant values.

Hazard ratios calculated using Cox proportional hazard model showed albumin level above 35 g/L to have a hazard of 0.58 ; 95% CI: 0.35 - 0.95; P = 0.03 (Table 4). Thirty day mortality rate in the hypo-albuminaemic group was 2% compared to 1% in the normo-albuminaemic group ( 95% CI: -3.4 - 5.4 ; P = 0.65). Post operative complication rates between the two groups were also not significantly different (albumin < 35 g/L - 22.9% vs. albumin > 35 g/L - 12.3%; 95% CI: - 2.2 - 23.3; P = 0.17).

**Table 4:** Hazard ratios calculated for significant determinants of survival using Cox-proportional hazard model

**Factor**	**DF**	**PE**	**Hazard ratio**	**95% CI**	**P value**
Circumferential margin positvity	1	0.79	**2.22**	1.26 - 3.89	0.005
Albumin level	1	-0.53	**0.58**	0.35 - 0.95	0.032
AJCC stage III/IV	1	0.70	**2.02**	1.26 - 3.23	0.003

## Discussion

The current study shows a significant association between a low preoperative serum albumin of less than 35 g/ L, with a reduction in overall survival for patients undergoing surgery for rectal cancer. Heys *et al.*, in the earliest available study of 481 patients with colon and rectal cancer, demonstrated a significant association between preoperative serum albumin and survival in large bowel cancer [16]. Since current evidence supports the observation that colon cancer is different to rectal cancer in terms of biological and clinicopathological characteristics [17,18] the authors feel that the two are best studied separately. A recent study on preoperative hypo-albuminemia in rectal cancer demonstrated its’ significant association with poor short term outcome only [13]. Lohsiriwat *et al.* in this study demonstrated a higher rate of overall postoperative complications and a longer hospital stay associated with hypoalbuminaemia, but did not report on overall survival. The current study did not show a statistical significant difference in the thirty day mortality and postoperative complication rate although both statistics were halved in the group with a serum albumin level of greater than 35 g/L. Taking colon cancer into consideration, some studies which reported predictability of survival using pre-operative albumin levels in serum, reported variable cut off values of albumin as a predictor of survival [18,19]. These studies have serum albumin level as a continuous variable. In the current study the highest significance with regard to overall survival was observed at an albumin level of 35 g/L when survival was compared with a univariable analysis based on serially ascending values of serum albumin. This corresponds to the lower limit of the standard range, reported in laboratories [20]. Roxburgh et al. in a review looking in to role of the systemic inflammatory response (SIR) in predicting survival for patients with cancer using biochemical or haematological markers recognised hypoalbuminaemia to be significant as an inflammatory marker along with C-reactive protein (CRP), neutrophils and lymphocyte/ platelet ratio [9]. A recent study by Carruthers et al. on patients with locally advanced rectal cancer concluded that the SIR is a predictor of overall and disease free survival outcome in those receiving neoadjuvant chemoradiation [14]. The authors have used neutrophil/ lymphocyte ratio as the indicator of inflammation. McMillan et al. in 2007 proposing the modified GPS reported a higher survival predictive value in CRP compared to hypoalbuminaemia in patients with Dukes stage B and C colorectal cancers [21]. Same group in a recent study assessing the outcome of the modified GPS in a lager cohort (n = 8759) reported that 90% of cancer patients with a low albumin level had an elevated CRP [22]. Therefore in a setting where serum albumin is assessed with routine liver profile, it appears to be a both cost effective and a sensitive marker to predict outcome given the fact that in a large majority the two markers correlate.

In the current study both overall survival and disease free survival in rectal cancer have shown to be adversely affected by preoperative hypoalbuminaemia. Therefore the evidence is accumulating to suggest a survival effect of the SIR in rectal cancer which can be predicted with easily accessed parameters such as albumin and white cell count. Furthermore, augmentation of serum albumin in the peri-operative period with albumin infusions has failed to demonstrate a significant benefit [23,24]. Serum albumin may be an indicator of a complex process involving tumour induced and pro-inflammatory factors that affect survival rather than a previously thought marker of nutritional status alone in these patients.

## Conclusion

Preoperative hypoalbuminaemia is a significant independent risk factor for poor overall and disease specific survival in rectal cancer. Given the available evidence, SIR seems to have a positive influence on the postoperative outcome in rectal cancer patients. There are multiple markers which could be used either alone or in combination as indicators of the SIR. Albumin can be used as a cost effective and a sensitive marker to predict survival in rectal cancer compared to other available inflammatory markers.

### Consent

Written informed consent was obtained from the patient for publication of this report and any accompanying images.

## Competing interests

The authors declare that they have no competing interests.

## Authors’ contribution

PCC was involves with conception and design, acquisition of data, analysis and interpretation of data, drafting the article and final approval of the version to be published. DSE was involved in conception and design, analysis and interpretation of data, revising the article critically for important intellectual content, and final approval of the version to be published. SK made a substantial contribution to conception and design, acquisition of data, revising the article critically for important intellectual content and final approval of the version to be published. KID was involved in conception and design, analysis and interpretation of data, drafting the article and revising it critically for important intellectual content. He was also involved in the final approval of the version to be published. All authors read and approved the final manuscript.

## Pre-publication history

The pre-publication history for this paper can be accessed here:

http://www.biomedcentral.com/1472-6890/13/12/prepub
